# Influence of ADAM10 Polymorphisms on Plasma Level of Soluble Receptor for Advanced Glycation End Products and The Association With Alzheimer’s Disease Risk

**DOI:** 10.3389/fgene.2018.00540

**Published:** 2018-11-13

**Authors:** Wen-Hui Huang, Wei Chen, Lian-ying Jiang, Yi-Xia Yang, Li-Fen Yao, Ke-Shen Li

**Affiliations:** ^1^Department of Neurology and Stroke Center, The First Affiliated Hospital of Jinan University, Guangzhou, China; ^2^Clinical Neuroscience Institute of Jinan University, Guangzhou, China; ^3^Guangdong Key Laboratory of Age-Related Cardiac and Cerebral Diseases, Affiliated Hospital of Guangdong Medical College, Zhanjiang, China; ^4^Department of Neurology, The First Affiliated Hospital, Harbin Medical University, Harbin, China

**Keywords:** Alzheimer’s disease, ADAM10, single nucleotide polymorphism (SNP), soluble receptor for advanced glycation end products, RAGE

## Abstract

To determine the role of A disintegrin and metalloproteinase 10 (ADAM10) in genetic susceptibility to Alzheimer’s disease (AD) in a representative Chinese sample, we genotyped 362 AD patients and 370 healthy controls for the rs514049A/C and rs653765C/T polymorphisms in the ADAM10 promoter using the SNaPshot technique. We also examined the potential impact of these polymorphisms on the plasma level of soluble receptor for advanced glycation end products (sRAGE), a decoy receptor whose reduction has been associated with a higher risk of AD. Additionally, a meta-analysis was performed using the present study and the largest GWAS from the International Genomics of Alzheimer’s Project (IGAP). No significant differences were found in the distributions of genotypes or alleles between AD patients and control subjects. However, age-at-onset stratification analysis revealed that there were significant differences in the genotypes (*P* = 0.015) and alleles (*P* = 0.006) of the rs653765 SNP. Furthermore, patients with the rs653765 CC genotype showed a lower ADAM10 level and a faster cognitive deterioration than those in patients with the CT/TT genotype in late-onset AD (LOAD), and the rs653765 CC polymorphism was able to regulate the production of the ADAM10 substrate sRAGE. In contrast, the rs514049 polymorphism was not statistically associated with AD. In the meta-analysis, we observed that both rs514049 (A allele vs. C allele, *P* = 0.002) and rs653765 (C allele vs. T allele, *P* = 0.004) were associated with AD risk. The present study indicated that the rs653765 polymorphism might be associated with the risk and development of LOAD; in particular, the risk genotype, CC, may decrease the expression of ADAM10, influencing the plasma levels of sRAGE, and thus may be correlated with the clinical progression of AD.

## Introduction

Alzheimer’s disease (AD), a progressive neurodegenerative disorder typically affecting older patients, is pathologically characterized by brain deposition of amyloid-β (Aβ) peptides as senile plaques and intracellular neurofibrillary tangles (Mattson, 2004). Aβ peptides of various lengths (e.g., Aβ40 and Aβ42) are generated in the amyloidogenic pathway by sequential cleavage of amyloid precursor protein (APP) through β- and γ-secretase. Fortunately, under nonpathological conditions, the vast majority of APP is constitutively processed within the nonamyloidogenic pathway by α- and γ-secretase activities. This cleavage releases a soluble, neuroprotective N-terminal ectodomain termed sAPPα and precludes the formation of pathogenic Aβ peptides (Kojro and Fahrenholz, 2005). Furthermore, Obregon et al. recently proved that sAPPα has the ability to mediate β-site APP-converting enzyme BACE1 inhibition and decrease Aβ pathologic generation (Obregon et al., 2012).

A disintegrin and metalloproteinase domain 10 (ADAM10), a member of the ADAM family, has been identified as the principal APP cleaving α-secretase. Accordingly, the important role that it plays in AD was manifested. A loss of ADAM10 levels has been detected in sporadic AD patients along with lower sAPPα levels (Colciaghi et al., 2002). An AD mouse model overexpressing ADAM10 revealed strongly attenuated plaque pathology and enhanced production of the α-secretase-derived soluble cleavage product sAPPα (Postina et al., 2004). Moreover, these mice had a clear improvement in memory and alleviation of learning deficits. In recent years, genetic association studies, especially large-scale genome-wide association studies (GWASs), have identified some novel AD risk genes (PICALM, CLU, CR1, BIN1, CD2AP, CD33, ABCA7, EPHA1, PLCG2, ABI3, TREM2) and risk pathways associated with the potential pathogenesis and genetic mechanisms of AD (Liu et al., 2012, 2013a,b, 2014a,b,c, 2017b, 2018; Lambert et al., 2013; Bao et al., 2015; Chen et al., 2015; Li et al., 2015, 2016; Shen et al., 2015; Xiang et al., 2015; Zhang et al., 2015, 2016; Jiang et al., 2017; Jun et al., 2017; Sims and van der Lee, 2017). However, a few studies have attempted to investigate the relationship between single nucleotide polymorphisms (SNPs) within the ADAM10 gene and AD risk. Kim et al. (2009) initially reported a genetic association between the ADAM10 rs2305421 polymorphism and AD in Caucasians. Song et al. replicated the study, and after stratifying the results by apolipoprotein E (ApoE) ε4 status, they found that the rs2305421 polymorphism within ADAM10 could be a risk factor for AD in a Chinese Han cohort (Song et al., 2011). In addition, ADAM10 promoter polymorphisms (rs514049 and rs653765) have been demonstrated to be associated with downregulated ADAM10 expression and sAPPα levels in cerebrospinal fluid (CSF) in both AD patients and healthy controls (Bekris et al., 2011, 2012), but an association between the polymorphisms and increased risk of AD in Caucasian populations was not found (Raucci et al., 2008; Bekris et al., 2012). A case-control study replicated in a Chinese Han cohort including 200 AD patients and 243 healthy participants also did not find an association between these polymorphisms and the risk of AD (Zeng et al., 2015).

The receptor for advanced glycation end products (RAGE), a multiligand receptor found in both neurons and cerebral microvascular endothelia that bind Aβ, is also a substrate for ADAM10 (Park et al., 2004; Emanuele et al., 2005). The interaction of RAGE with Aβ has been implicated in the amplification of oxidative stress, mitochondrial dysfunction and inflammation, resulting in RAGE-induced Alzheimer-like pathophysiological changes that contribute to the development of AD (Cai et al., 2016). Ectodomain shedding of RAGE by ADAM10 generates a soluble counterpart of RAGE, sRAGE, which lacks the cytosolic and transmembrane domains. This observation is of particular interest because ligand-induced RAGE signaling is involved in AD pathological processes, while sRAGE acts as a decoy receptor that antagonizes RAGE-mediated adverse effects (Park et al., 2004; Yan et al., 2009). Recently, the expression level of circulating sRAGE was reported to be downregulated in the plasma of AD patients (Xu et al., 2017). Moreover, in our previous study, we demonstrated that the RAGE G82S variant reduced the plasma level of sRAGE and was associated with an increased risk of AD (Li et al., 2010). It is noted that the presence of sRAGE in biological fluids could affect the function of RAGE ligands by competing for ligand binding with membrane-bound RAGE. Thus, by preventing the interaction of ligands with cell surface RAGE, sRAGE can exert significant protective effects against RAGE-mediated toxicity.

Given the key role of ADAM10 in shifting cell surface RAGE to sRAGE, we tested the hypothesis that genetic variations in the ADAM10 promoter region might affect the expression and function of ADAM10 and then regulate the availability of cell surface-localized RAGE and its soluble ectodomain. Therefore, we conducted a hospital-based case-control association study in Chinese AD patients to investigate the following unresolved issues: (1) whether these two functional promoter polymorphisms of the ADAM10 gene are associated with the plasma level of sRAGE in AD and (2) whether these two polymorphisms affect the risk of AD.

## Materials and Methods

### Subject Characteristics

The demographic details and clinical features of the two groups are shown in Table 1. Our study consecutively enrolled 362 AD patients (118 males and 244 females; mean age = 72.8 ± 8.1 years) and 370 healthy controls (168 males and 202 females; mean age = 74.2 ± 7.7 years) from the Department of Neurology at the First Affiliated Hospital of Jinan University and the Affiliated Hospital of Guangdong Medical University. The age, sex and clinical features were homogeneous between the two groups. As reported, more ApoE ε4 carriers were detected in the AD group than in the control group (*P* < 0.05). The control group was confirmed to be healthy and neurologically normal by medical history, general examinations, laboratory examinations and the Mini Mental State Examination (MMSE) (score > 28). Clinical diagnosis of probable AD was performed according to the criteria of the National Institute of Neurological and Communicative Disorders and Stroke and the Alzheimer’s Disease and Related Disorders Association (NINCDS-ADRDA) and DSM-III-R criteria (McKhann et al., 1984). All cases were reviewed by two neurologists, and a final consensus diagnosis was established in each case. All patients were defined as sporadic because dementia did not exist among their first-degree relatives in their family history. Subjects with myocardial infarction, congestive heart failure, stroke, type 2 diabetes mellitus (T2DM) and atherosclerosis were excluded from this study. All participants included in this study were unrelated Chinese Han individuals. Other forms of dementia were excluded from our study by using a standardized battery of examinations, including psychiatric interviews, general medical history, neurological examinations and neuropsychological testing. Patients whose age was ≤65 years were defined as early onset AD (EOAD; 64 patients), while those aged >65 years were defined as late-onset AD (LOAD; 298 patients). This study was carried out in accordance with the Institutional Review Board of the First Affiliated Hospital of Jinan University. All participants gave written informed consent in accordance with the Declaration of Helsinki.

**Table 1:** Characteristics of subjects in the AD and control groups.

Clinical features	AD (*n* = 362)	Controls (*n* = 370)
Age (years)	72.8 ± 8.1	74.2 ± 7.7
Sex (male: female)	118:244	168:202
Mean disease duration (years: range)	3.1 (0.7–16)	
>65 years	298/362	289/370
≤65 years	64/362	81/370
ApoE ε4 carriers	138/362	47/370
ApoE ε4 noncarriers	224/362	323/370

A subgroup including 178 LOAD patients was followed up for 2 years, and cognitive performances were recorded. Longitudinal cognitive decline was assessed by MMSE scores according to a previously published method in the literature (Doody et al., 2001). Briefly, LOAD patients were divided into three groups with different rates of deterioration (fast = decline of more than five points/year; intermediate = 2–4.9 points/year; slow = less than 2 points/year).

### Genotyping

Peripheral blood samples were collected from each subject, and genomic DNA was then extracted from peripheral blood with a Whole Blood Genomic DNA Blood Isolation Kit (Sangon Biotech, Shanghai, China) according to the manufacturer’s instructions. The purified DNA was quantified with a spectrophotometer and temporarily stored at -80°C prior to genotyping. Genotyping was performed for polymorphisms in ADAM10 (rs514049 and rs653765) using a SNaPshot Multiplex Kit (Genesky Biotechnologies, Inc., Shanghai, China). For 514049, the forward primer was AGCACCTCCCTCTCGCTCCAC, and the reverse primer was TTTTTTTTTTTTTTTTTTTAAGAAGAAAAAAAACCTCTGTTACTTGTGAC. For rs653765, the forward primer was AGCACCTCCCTCTCGCTCCAC, and the reverse primer was TGAGGCGGAGGTCTGAGTTTCGA. Polymerase chain reaction (PCR) was carried out in a final volume of 10 μL, which contained 5 μL of the SNaPshot Multiplex Kit reagent, 2 μL of the templates containing the multiplex PCR products, 1 μL of the primer mix and 2 μL of water. The PCR program was as follows: initial denaturation at 95°C/1 min; then denaturation at 94°C/10 s, annealing at 52°C/5 s, and a final extension at 60°C/30 s for a total of 28 cycles. The amplified products were stored at 4°C. The extension products were then purified for 1 h at 37°C with the assistance of shrimp alkaline phosphatase, followed by incubation for 15 min at 75°C to inactivate this enzyme. After purification, the purified products were analyzed using an ABI 3730XL DNA Analyzer and GeneMapper 4.1 (Applied Biosystems, Carlsbad, CA, United States). In addition, 5% of the samples were randomly selected as the validation group for regenotyping in an independent trial. ApoE genotyping was performed as described by Donohoe et al. (1999).

### Enzyme-Linked Immunosorbent Assay (ELISA)

Blood samples from AD patients (*n* = 110) and healthy controls (*n* = 105) were collected into EDTA-containing tubes and centrifuged at low speed, and the plasma aliquots were stored at -20°C. We performed ELISA to measure the level of sRAGE in three technical replicates using an ELISA kit (R&D Systems, Minneapolis, MN, United States) according to the manufacturer’s instructions. Then, the absorbance of each sample was read at 450 nm using a microplate reader, and the sRAGE levels were validated according to a standard curve.

### Mononuclear Cell Isolation and RNA Extraction

Peripheral blood mononuclear cells (PBMCs) are chiefly lymphocytes and monocytes. Human PBMCs were separated by density gradient centrifugation using Ficoll Histopaque (Sigma) performed as described previously. Total RNA was extracted from PBMCs using an RNAprep Pure Blood Kit (TianGen) as recommended by the manufacturer. In brief, PBMCs were lysed in TRIzol for 5 min at room temperature and centrifuged at 12,000 rpm for 10 min at 4°C. The aqueous phase was transferred to a fresh microcentrifuge tube, mixed with an equal volume of isopropanol and incubated for 25 min at room temperature. RNA was precipitated by centrifugation at 12,000 rpm for 10 min at 4°C. The RNA pellet was washed with 75% RNase-free ethanol and dissolved in RNase-free H_2_O. Then, the concentration and purity of the RNA samples were determined using a Bioanalyzer system (Thermo Fisher Scientific, Waltham, MA, United States). The integrity of RNA was confirmed by agarose gel electrophoresis, and the samples were stored at -80°C.

### Real-Time PCR

A First Strand cDNA Synthesis Kit (Invitrogen Life Technologies) was used to reverse transcribe first-strand cDNA from samples with an equal amount of RNA according to the manufacturer’s instructions. The mRNA expression level of ADAM10 was measured via quantitative real-time PCR using a SYBR green method and normalized to the level of housekeeping gene glyceraldehyde-3-phosphate dehydrogenase (GAPDH). The primers used in the assay were as follows: ADAM10 sense primer, CTGGCCAACCTATTTGTGGAA; ADAM10 antisense primer, GACCTTGACTTGGACTGCACTG; GAPDH sense primer, GAAGGGCTCATGACCACAGTCCAT; GAPDH antisense primer, TCATTGTCGTACCAGGAAATGAGCTT. In brief, PCR amplification was carried out in a 10 μL final volume containing 5 μL of 2× SYBR Green PCR master mix (TaKaRa), 0.2 μL of each specific forward and reverse primer, 3.6 μL of DNase-free water, and 1 μL of cDNA as a template. The real-time PCR program was as follows: 95°C for 30 s and 40 cycles of 95°C for 5 s and 62°C for 20 s. Each sample was analyzed in triplicate, and the averaged threshold cycle (CT) values of each reaction were calculated. The fold change in ADAM10 relative expression was then obtained using the 2^-ΔΔCT^ method.

### Meta-Analysis

We further performed a meta-analysis of our study and the largest GWAS from the International Genomics of Alzheimer’s Project (IGAP) (Lambert et al., 2013). The IGAP stage 1 genotyped and inputted 7,055,881 SNPs from 17,008 AD patients and 37,154 controls (Lambert et al., 2013). Here, we selected the allele model. We first selected the Cochran’s Q method to test the potential heterogeneity. Then we used a fixed effect model (Mantel-Haenszel) or a random-effect model (DerSimonian-Laird) to perform the meta-analysis, which is determined by the heterogeneity test (Li et al., 2016). The significance of meta-analysis is determined by *Z*-test. All statistical tests for heterogeneity and meta-analysis were computed using R Package^1^. More detailed meta-analysis methods have been widely described in previous studies (Liu et al., 2013b, 2017a,b, 2018; Hu et al., 2017a,b).

### Statistical Analysis

Differences in the characteristics of the subjects between the AD and control groups were compared by Student’s t-test or a chi-square test. Hardy-Weinberg equilibrium was tested using a chi-square test. Power analysis was performed with Quanto 1.2 software (CA, United States) under a given sample size and significance level of 0.05. Allele frequencies and genotype distributions were obtained by direct counting, and allele frequencies were calculated from the genotypes of all of the subjects. Allelic and genotypic distributions between the groups were performed using a chi-square test and Fisher’s exact test when appropriate. Odds ratios (OR) were calculated along with 95% confidence intervals (CI) to assess the relative risk. ADAM10 genotype data were corrected for the influence of age and gender by logistic regression, and the adjusted OR was then assessed. Plasma sRAGE levels were compared using the Kruskal-Wallis test and Student’s *t*-test between independent groups. All statistical analyses were performed using SPSS 21.0 software. Statistical significance was considered at a *P* < 0.05.

## Results

The genotype and allele frequencies for the ADAM10 promoter polymorphisms in patients with AD and controls are presented in Tables 2, 3. The distribution of the rs514049 and rs653765 genotypes in the AD patients and controls did not deviate from those predicted by Hardy-Weinberg equilibrium. Based on our sample size, power analysis showed that this study had 97.0% power for rs514049 and 87.8% power for rs653765 to detect a genotype with an OR of 1.7 at a significance level of 0.05. No significant differences in the genotype or allele frequencies were observed between the AD patients and the controls. After stratifying AD patients by the presence or absence of ApoE ε4, we did not observe any association between the gene promoter polymorphisms and AD.

**Table 2:** Frequency distribution of rs514049 genotypes and alleles in Alzheimer’s disease patients and healthy controls.

		Genotypes n (%)					Alleles n (%)				
rs514049	n (%)	AA	AC	CC	*P*	OR (95% CI)	A	C	*P*^∗^	OR^∗^ (95% CI)
**AD**		
Total	362 (49.5)	306 (84.5)	52 (14.4)	4 (1.1)	0.879	0.97 (0.66, 1.45)	664 (91.7)	60 (8.3)	0.837	0.96 (0.65, 1.42)
ApoE varepsilon4 (+)	138 (38.1)	115 (83.3)	21 (15.2)	2 (1.5)	0.349	0.62 (0.23, 1.70)	251 (90.9)	25 (9.1)	0.220	0.66 (0.34, 1.28)
ApoE varepsilon4 (-)	224 (61.9)	191 (85.3)	31 (13.8)	2 (0.9)	0.779	1.07 (0.67, 1.72)	413 (92.2)	35 (7.8)	0.694	1.09 (0.71, 1.67)
≤65 years	64 (17.7)	52 (81.2)	11 (17.2)	1 (1.6)	0.792	0.88 (0.31, 2.07)	115 (89.9)	13 (10.1)	0.899	0.95 (0.43, 2.10)
>65 years	298 (82.3)	254 (85.2)	41 (13.8)	3 (1.0)	0.929	0.98 (0.64, 1.55)	549 (92.1)	47 (7.9)	0.833	0.95 (0.59, 1.53)
Male	118 (32.6)	99 (83.9)	18 (15.3)	1 (0.8)	0.744	0.90 (0.48, 1.70)	216 (91.5)	20 (8.5)	0.765	0.91 (0.49, 1.69)
Female	244 (67.4)	207 (84.8)	34 (13.9)	3 (1.3)	0.692	1.10 (0.69, 1.77)	448 (91.8)	40 (8.2)	0.629	1.14 (0.67, 1.94)
**Controls**		
Total	370 (50.5)	315 (85.1)	50 (13.5)	5 (1.4)			680 (91.9)	60 (8.1)		
ApoE varepsilon (+)	47 (12.7)	42 (89.4)	4 (8.5)	1 (2.1)			88 (93.6)	6 (6.4)		
ApoE varepsilon4 (-)	323 (87.3)	273 (84.5)	46 (14.2)	4 (1.3)			592 (91.6)	54 (8.4)		
≤65 years	81 (21.9)	67 (82.7)	12 (14.8)	2 (2.5)			146 (90.1)	16 (9.9)		
>65 years	289 (78.1)	248 (85.8)	38 (13.2)	3 (1.0)			534 (92.4)	44 (7.6)		
Male	168 (45.4)	144 (85.7)	21 (12.5)	3 (1.8)			309 (92.0)	27 (8.0)		
Female	202 (54.6)	171 (84.7)	29 (14.4)	2 (0.9)			371 (91.8)	33 (8.2)		

*OR, odds ratio; CI, confidence interval. P, P^∗^ value and OR (95% CI) have been adjusted for gender and age. P-value, OR for AA and AC + CC genotypes; P^∗^ value, OR^∗^ for A and C alleles.*

**Table 3:** Frequency distribution of rs653765 genotypes and alleles in Alzheimer’s disease patients and healthy controls.

	Genotypes n (%)					Alleles n (%)				
rs653765	n (%)	CC	CT	TT	*P*	OR (95% CI)	C	T	*P*^∗^	OR^∗^(95% CI)
**AD**		
Total	362 (49.5)	276 (76.2)	77 (21.3)	9 (2.5)	0.188	1.25 (0.90, 1.75)	629 (86.9)	88 (13.1)	0.174	1.27 (0.90, 1.79)
ApoE varepsilon4 ( + )	138 (38.1)	106 (76.8)	30 (21.7)	2 (1.5)	0.919	1.04 (0.50, 2.26)	232 (87.7)	34 (12.3)	0.384	1.33 (0.70, 2.53)
ApoE varepsilon4 (-)	224 (61.9)	170 (75.9)	47 (21.0)	7 (3.1)	0.158	1.32 (0.87, 1.88)	387 (86.4)	61 (13.6)	0.283	1.20 (0.86, 1.67)
≤ 65 years	64 (17.7)	49 (76.6)	13 (20.3)	2 (3.1)	0.115	0.49 (0.20, 1.18)	111 (86.7)	17 (13.3)	0.116	0.52 (0.23, 1.18)
> 65 years	298 (82.3)	227 (76.2)	64 (21.5)	7 (2.3)	0.015	1.55 (1.14, 2.31)	518 (86.9)	78 (13.1)	0.006	1.54 (1.13, 2.10)
Male	118 (32.6)	92 (78.0)	20 (17.0)	6 (5.0)	0.256	1.38 (0.79, 2.40)	204 (86.4)	32 (13.6)	0.542	1.16 (0.72, 1.87)
Female	244 (67.4)	184 (75.4)	57 (23.4)	3 (1.2)	0.330	1.23 (0.80, 1.85)	425 (87.1)	79 (12.9)	0.855	1.03 (0.75, 1.41)
**Controls**		
Total	370 (50.5)	265 (71.6)	93 (25.1)	12 (3.3)			623 (84.2)	117 (15.8)		
ApoE varepsilon4 (+)	47 (12.7)	36 (76.6)	7 (14.9)	4 (8.5)			79 (84.0)	15 (16.0)		
ApoE varepsilon4 (-)	323 (87.3)	229 (70.9)	86 (26.6)	8 (2.5)			544 (84.2)	102 (15.8)		
≤65 years	81 (21.9)	71 (87.7)	9 (11.1)	1 (1.2)			151 (93.2)	11 (6.8)		
>65 years	289 (78.1)	194 (67.1)	84 (29.1)	11 (3.8)			472 (81.7)	106 (18.3)		
Male	168 (45.4)	120 (71.4)	43 (25.6)	5 (3.0)			283 (84.2)	53 (15.8)		
Female	202 (54.6)	145 (71.8)	50 (24.8)	7 (3.4)			340 (84.2)	64 (15.8)		

*OR, odds ratio; CI, confidence interval. P, P^∗^ value and OR (95% CI) have been adjusted for gender and age. P value, OR for CC and CT + TT genotypes; P^∗^ value, OR^∗^ for C and T alleles.*

AD patients were then divided into EOAD and LOAD, and controls were also stratified according to age (≤65 years or >65 years). The ADAM10 promoter rs653765 genotype distribution in the study participants is presented in Table 3. The frequencies of the CC genotype and C allele were higher in LOAD patients than in control subjects older than 65 years (76.2% vs. 67.1%, OR = 1.55, 95% CI: 1.14–2.31, and *P* = 0.015; 86.9% vs. 81.7%, OR = 1.54, 95% CI: 1.13–2.10, and *P* = 0.006, respectively), however, no statistically significant differences in genotype distribution were observed between EOAD patients and controls younger than 65 years (*P* = 0.115). In contrast, there were no statistically significant differences in the genotypes of the ADAM10 rs514049 promoter polymorphism between the AD patients and the healthy subjects (*P* = 0.879). Taken together, these results suggest that the rs653765 CC genotype and C allele are likely risk factors for LOAD. Stratifying AD patients and controls by gender showed no significant difference (rs514049 and rs653765) in phenotype and allele frequencies between AD patients and controls in either males or females.

The ADAM10 rs514049 and rs653765 genotype distributions were also investigated in a subgroup (*n* = 178) of patients clinically diagnosed with LOAD followed up for 2 years and assessed for cognitive performance. Patients were divided into three cohorts according to the rate of cognitive deterioration (fast, intermediate, and slow) and stratified by ADAM10 polymorphism genotypes (Table 4). We did not find a significant difference in rs514049 polymorphisms in LOAD patients (χ^2^ = 0.228, *P* = 0.892). However, an increased representation of the ADAM10 CC genotype was observed in LOAD patients with fast cognitive deterioration (78.6%) compared with that in patients with slow deterioration rates (55.1%) (χ^2^ = 7.167, *P* = 0.028).

**Table 4:** Genotype distribution of the rs653765 polymorphism in LOAD patients stratified according to the rate of cognitive decline.

Rate of cognitive decline	rs514049 Genotypes n (%)		rs653765 Genotypes n (%)	
	AA	AC + CC	CC	CT + TT
Fast (*n* = 42)	35 (83.3)	7 (16.7)	33 (78.6)	9 (21.4)
Intermediate (*n* = 67)	54 (80.6)	13 (19.4)	47 (70.1)	20 (29.9)
Slow (*n* = 69)	55 (79.7)	14 (20.3)	38 (55.1)	31 (44.9)

*rs514049: χ^*2*^ = 0.228, P = 0.892; rs653765: χ^*2*^ = 7.167, P = 0.028.*

**FIGURE 1:**
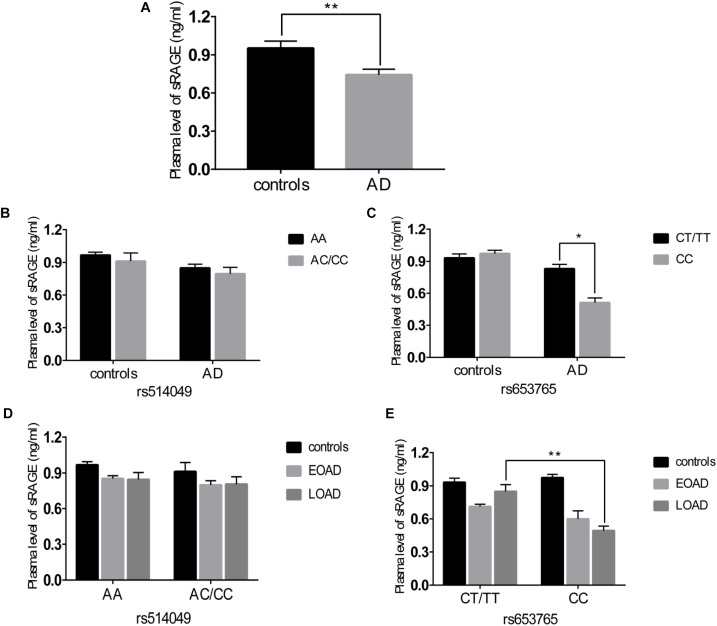
Plasma soluble RAGE (sRAGE) levels in AD patients and healthy controls. **(A)** Plasma sRAGE levels in AD patients and healthy controls. Plasma sRAGE levels by different genotypes of rs514049 **(B)** and rs653765 **(C)** in AD patients and healthy controls. Plasma sRAGE levels by different genotypes of rs514049 **(D)** and rs653765 **(E)** in healthy controls, early onset AD (EOAD) and late-onset AD (LOAD). Data are presented as the means ± SEM. ^∗^*P* < 0.05; ^∗∗^*P* < 0.01.

**FIGURE 2:**
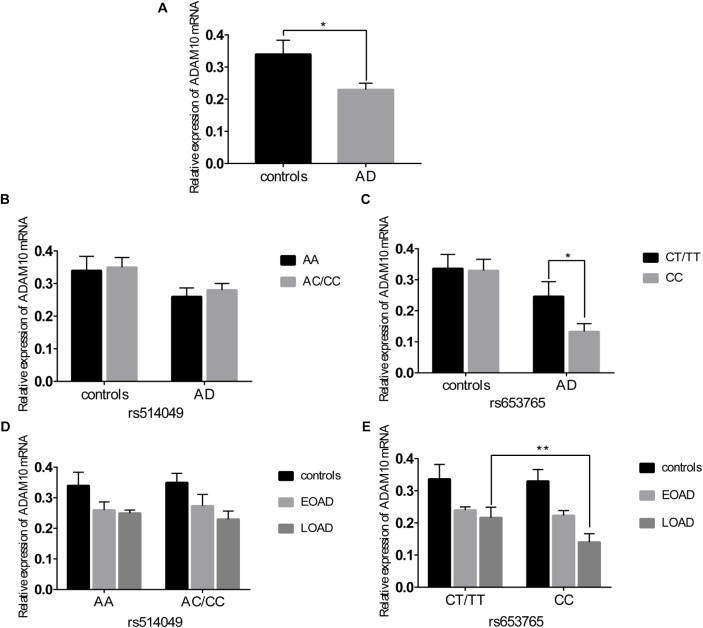
Expression level of A disintegrin and metalloproteinase 10 (ADAM10) in AD patients and healthy controls. **(A)** Expression level of ADAM10 in AD patients and healthy controls. Expression level of ADAM10 by different genotypes of rs514049 **(B)** and rs653765 **(C)** in AD patients and healthy controls. Expression level of ADAM10 by different genotypes of rs514049 **(D)** and rs653765 **(E)** in healthy controls, early onset AD (EOAD) and late-onset AD (LOAD). Data are presented as the means ± SEM. ^∗^*P* < 0.05; ^∗∗^*P* < 0.01.

A comparative determination was performed at the level of ADAM10 and sRAGE in 110 patients with AD and 105 controls. According to the data obtained, we observed that the mean value of the plasma sRAGE level was significantly downregulated in the AD cases compared with that of the controls (Figure 1A). Furthermore, we also analyzed whether there was an association between the mean value of the plasma sRAGE level and different ADAM10 promoter genotypes. A significant decrease in plasma sRAGE levels was observed in patients with AD who carried the rs653765 CC genotype, and the differences were statistically significant (Figure 1C). Stratifying AD patients and healthy controls according to age of onset showed that the subjects with the CC genotype exhibited lower plasma sRAGE levels compared to subjects with the CT/TT genotypes, and these differences were statistically significant (Figure 1E). No such significant differences in the sRAGE level were observed for the rs514049 polymorphism (Figures 1B,D). Interestingly, our results showed the same decreasing trend in the mRNA expression level of ADAM10 for the rs653765 polymorphism (Figure 2). The relative expression of ADAM10 mRNA was significantly downregulated in the AD cases compared with that of the controls (Figure 2A). Those AD patients who carried the CC phenotype of ADAM10 had lower levels of ADAM10 than those carrying the CT/TT genotypes (Figures 2C,E). For the rs514049 polymorphism, no statistically significant difference in ADAM10 level was observed between the AD patients carrying the rs514049 mutated genotype and those carrying the nonmutated genotype (Figures 2B,D).

In the meta-analysis derived from IGAP dataset and the present association study, we got the summary results of rs514049 and rs653765. In brief, both rs514049 (A allele vs. C allele, beta = -0.049, se = 0.016, and *P* = 0.002) and rs653765 (C allele vs. T allele, beta = -0.051, se = 0.018, and *P* = 0.004) were associated with AD risk. For rs514049, Cochran’s Q test did show significant heterogeneity with Iˆ2 = 0%, and *P* = 0.966. Meta-analysis of our data and IGAP dataset showed that rs514049 was significantly associated with AD risk using both fixed effect model (OR = 0.95, 95% CI: 0.93–0.98, and *P* = 0.002) and a random-effect model (OR = 0.95, 95% CI: 0.93–0.98, and *P* = 0.002). For rs653765, Cochran’s Q test showed evidence of heterogeneity with Iˆ2 = 62.9%, and *P* = 0.100. Meta-analysis of our data and IGAP dataset showed that rs653765 was significantly associated with AD risk using the fixed effect model (OR = 0.95, 95% CI: 0.92–0.99, and *P* = 0.006), but not a random-effect model (OR = 1.04, 95% CI: 0.80–1.36, and *P* = 0.760).

## Discussion

In our case-control study including 362 AD patients and 370 control subjects, we found no statistically significant differences in the genotype or allele frequencies between AD cases and controls, suggesting that the ADAM10 promoter polymorphisms may not be risk factors for the occurrence of AD. However, when these data were stratified by age at onset, we found that individuals carrying the ADAM10 rs653765 CC genotype confirmed the risk of this genetic variant in LOAD patients. Indeed, analysis of a cohort including 110 AD patients and 105 healthy controls indicated that participants carrying the ADAM10 rs653765 CC genotype expressed lower ADAM10 mRNA levels than individuals carrying the CT/TT genotypes. Importantly, the rs653765 CC genotype carriers exhibited a lower plasma sRAGE level than CT/TT genotype carriers among AD patients, suggesting that rs653765 C allele carriers might be less competent at antagonizing cell surface RAGE and thereby may be more susceptible to the Aβ-induced cellular perturbation than carriers of the wild-type allele. To the best of our knowledge, this is the first study to investigate the influence of ADAM10 promoter polymorphisms on the plasma level of sRAGE and its association with AD risk in the Chinese Han population.

There is emerging evidence of the critical role of ADAM10 in the pathogenic mechanisms leading to AD. Changes in the expression of ADAM10 have been detected in AD patients. Gatta et al. reported that the mRNA expression level of ADAM10 was elevated in severe cases of AD (Gatta et al., 2002), whereas Colciaghi et al. (2002) observed decreased ADAM10 and sAPPα levels in human platelets and CSF of AD patients. Despite these contradictory observations, overexpression of ADAM10 has been shown to alleviate the production of Aβ and have a protective effect in an AD mouse model (Park et al., 2004; Kuang et al., 2017; Volmar et al., 2017). It has been previously demonstrated that vitamin A and its analog can affect the transcriptional activity of the ADAM10 promoter and enhance the nonamyloidogenic sAPPα secretion pathway, which may act as a potential therapeutic target for AD (Tippmann et al., 2009). To date, few studies have attempted to analyze the association of ADAM10 gene polymorphisms with the genetic susceptibility to AD. Kim et al. discovered two rare, partially penetrant, familial late-onset AD mutations in the ADAM10 gene that led to attenuated α-secretase activity and determined ADAM10 to be a candidate AD susceptibility gene (Kim et al., 2009). However, a large-scale (*n* = 576: controls, 271; AD, 305) resequencing study performed by Cai et al. found no significant association between ADAM10 mutations and AD risk (Cai et al., 2012). In addition, a recent independent study genotyping 27 SNPs covering the entire ADAM10 gene in a larger cohort of patients revealed no single-marker or haplotypic association with AD risk (Laws et al., 2011). Further studies are certainly needed to clarify the relationship between ADAM10 gene polymorphisms and AD.

Recently, it has been demonstrated that nucleotides -2179 to -1 upstream of the ADAM10 gene represented a functional TATA-less promoter and identified several SNPs in the promoter region of ADAM10. Bekris et al. (2011, 2012) found that the ADAM10 promoter polymorphisms (rs514049 and rs653765) were not associated with AD risk in a Caucasian population. Zeng et al. (2015) replicated the ADAM10 rs514049 and rs653765 polymorphism analysis in a Chinese Han cohort including 200 AD patients and 243 controls and did not find an association between ADAM10 polymorphisms and the risk of AD. In our study, there were no statistically significant differences in the genotype distributions of the rs514049 and rs653765 polymorphisms between overall AD patients and healthy controls (OR = 0.97, 95% CI = 0.66–1.45, and *P* = 0.879; OR = 1.25, 95% CI = 0.90–1.75, and *P* = 0.188, respectively). These findings were partly in agreement with the results from a study conducted in a Chinese Han cohort (Zeng et al., 2015). However, unlike the previous case-control study, our results indicated a significant difference in the rs653765 CC genotype and C allele between the individuals with LOAD and controls older than 65 years when these data were stratified by the age at onset. A logistic regression analysis adjusted for age and gender still showed that the CC genotype and C allele seemed to indicate an increased risk of LOAD (OR = 1.55, 95% CI = 1.14–2.31, and *P* = 0.015; OR = 1.54, 95% CI = 1.13–2.10, and *P* = 0.006, respectively), while the rs514049 polymorphism of ADAM10 was negatively associated with LOAD. Meta-analysis of our data and IGAP dataset showed that rs514049 was significantly associated with AD risk using both fixed effect model (OR = 0.95, 95% CI = 0.93–0.98, and *P* = 0.002) and a random-effect model (OR = 0.95, 95% CI: 0.93–0.98, and *P* = 0.002). For rs653765, these results showed that rs653765 was significantly associated with AD risk using the fixed effect model (OR = 0.95, %95 CI: 0.92–0.99, and *P* = 0.006), but not a random-effect model (OR = 1.04, 95% CI: 0.80–1.36, and *P* = 0.760). The data demonstrate that the rs514049 C allele and rs653765 allele may affect susceptibility to AD. Here, the rs514049 allele frequency was 91.7% for the A allele, and the rs653765 allele frequency was 86.9% for the C allele, similar to previous data in Chinese individuals (93.6% for the rs514049 A allele and 88.0% for the rs653765 C allele) (Cui et al., 2015). However, these allele frequencies were significantly different from another study of white individuals whose rs514049 A allele frequency was 39.8% and rs653765 C allele frequency was 24.1% (Armstrong et al., 2007). This discrepancy might result from profound ethnic differences.

RAGE, a multiligand receptor of the immunoglobulin superfamily, functions as a signal-transducing cell surface acceptor for Aβ (Yan et al., 1996; Srikanth et al., 2011). Increased expression of RAGE is observed in regions of the brain affected by AD, and the Aβ-RAGE interaction mediates Aβ neurotoxicity, promotes Aβ influx into the brain and contributes to Aβ aggregation. Fortunately, RAGE is subject to ectodomain shedding by ADAM10, and the derived sRAGE can inhibit the Aβ-RAGE interaction, block Aβ influx across the BBB and alleviate RAGE-mediated cellular perturbation in AD (Zhang et al., 2009). Although several groups have shown that sRAGE is derived from alternative splicing of RAGE mRNA, recent studies demonstrated that the major pathway for the production of circulating sRAGE appears to be enzymatic cleavage of full-length RAGE from the plasma membrane by the enzyme ADAM10. In this study, we found that individuals carrying the rs653765 CC genotype expressed lower ADAM10 mRNA levels compared with those carrying the CT/TT genotype. Importantly, the ADAM10 rs653765 CC genotype carriers exhibited a lower plasma sRAGE level than CT/TT genotype carriers among AD patients, suggesting that rs653765 C allele carriers might be less competent at antagonizing cell surface RAGE and thereby may be more susceptible to Aβ-induced cellular perturbation than carriers of the wild-type allele. In addition, we also demonstrated a significant reduction in plasma sRAGE levels in AD patients compared with those in controls, which provides a clinically significant explanation for detecting plasma sRAGE levels in evaluating the risk of AD. Given the recent identification of ADAM10 as the sheddases for RAGE, it is conceivable that the decrease in plasma sRAGE may be due to the decrease in either ADAM10 expression or ADAM10 activity in AD patients. Consistent with this concept, Emanuele et al. discovered that the plasma level of sRAGE was significantly decreased in AD patients compared with controls, which may act as a biological marker for AD (Emanuele et al., 2005). Moreover, our previous study proved that the RAGE G82S polymorphism was associated with a decrease in plasma sRAGE concentration, which potentially contributed to the elevated risk in AD patients (Li et al., 2010). We also found a significant decrease in sRAGE levels in LOAD patients carrying the rs653765 CC genotype compared with those carrying the CT/TT genotype, while this significant difference was not observed in EOAD patients or healthy controls. It was previously reported that sRAGE has a protective effect on amyloid fibrillogenesis. As observed by atomic force microscopy, even with long periods of coincubation, sRAGE significantly abolished further aggregation of Aβ fibrillogenesis (Chaney et al., 2005). Thus, lower plasma levels of sRAGE in AD patients carrying the rs653765 C allele might further favor Aβ fibril formation, thereby leading to a further decline in sRAGE in LOAD. Taken together, these findings indicate that the circulating level of sRAGE in AD appears to be correlated with the severity of the disease, in accordance with previous findings.

Notably, considering the association between the rs653765 polymorphism and cognitive deterioration, the CC genotype was associated with an increased risk of fast deterioration. It was reported that RAGE is a key cofactor for Aβ-mediated cellular perturbation relevant to the cognitive impairment of AD (Yan et al., 2012). Double transgenic mice overexpressing neuronal RAGE and mAPP displayed earlier onset of spatial learning/memory function abnormalities compared to animals expressing only mutant APP (Arancio et al., 2004). Furthermore, sRAGE acts as a decoy that binds to RAGE ligands to inhibit the RAGE-Aβ interaction and prevent the adverse effects of RAGE signaling. Emerging findings suggest a reduced level of plasma sRAGE in patients with AD or mild cognitive impairment (MCI) (Emanuele et al., 2005; Ghidoni et al., 2008). In addition, levels of sRAGE in the plasma are associated with the level of cognitive impairment in AD and MCI patients (Hernanz et al., 2007). Thus, it is reasonable to speculate that in patients carrying the CC genotype, an upregulation of RAGE-mediated intracellular signaling, consistent with a reduction in plasma sRAGE levels, may give rise to a fast-cognitive deterioration rate.

In the present study, we discovered a positive correlation of the rs653765 polymorphism with sRAGE serum concentrations, while no such association of the ADAM10 gene rs514049 polymorphism with sRAGE was detected. Thus, our results demonstrated that the CC genotype of the rs653765 polymorphism in the ADAM10 gene may contribute to the genetic susceptibility to LOAD in Han Chinese populations. Its presence correlates with a lower level of sRAGE, whose downregulation represents a known risk factor for AD. The potential mechanism by which this genotype contributes to the risk for the development of AD could be mediated by a decrease in sRAGE, however, the results of the case-control analysis in the EOAD/LOAD or ApoE subgroups remain preliminary due to the small number of subjects, and further studies with a larger cohort in different populations are required.

## Author Contributions

K-SL conceived and designed the experiments. W-HH performed the experiments and wrote the paper. W-HH, WC, and L-FY analyzed the data. L-yJ and Y-XY helped to collect all the samples and analyze statistical data. All authors read and approved the final manuscript.

## Conflict of Interest Statement

The authors declare that the research was conducted in the absence of any commercial or financial relationships that could be construed as a potential conflict of interest.

## Footnotes

1 http://cran.r-project.org/web/packages/meta/index.html
